# Sn- and Mo-Modified Sulfonated Carbons: Properties and Evaluation as Catalysts for Fructose Conversion in Water and DMSO

**DOI:** 10.1021/acsomega.5c00450

**Published:** 2025-05-29

**Authors:** Felyppe Markus Ribeiro Sobral Altino, Wander dos Santos Sá, Jailma Barros dos Santos, Wagner Alves Carvalho, Simoni Margareti Plentz Meneghetti

**Affiliations:** † Group of Catalysis and Chemical Reactivity (GCAR), Institute of Chemistry and Biotechnology, 28112 Federal University of Alagoas, Maceió, AL 57072-970, Brazil; ‡ Centre for Natural Sciences and Humanities, 74362 Federal University of ABC (UFABC), Santo André, SP 09280-560, Brazil

## Abstract

Sulfonated carbon-based materials produced from residual glycerol from biodiesel were modified with metallic species (Sn and Mo) to modulate their acidic properties. The materials C, CSn3, CMo3, and CSn3Mo3 presented surface areas of 46.8, 29.3, 67.7, and 43.8 m^2^ g^–1^, respectively. The presence of Sn and Mo, which impart Lewis acidity to the systems, can be evidenced by ICP-OES and XRD, while the presence of groups acting as Brønsted acids is clearly observed through FTIR. Their application as heterogeneous catalysts for fructose conversion in water or DMSO revealed that varying the Sn content had a minimal effect on the conversion rates. However, since the CSn3 system stood out for presenting slightly better performance despite having the lowest Sn content among the tested materials (conversion of 41.8% after 6 h), it was chosen to be modified with Mo. The incorporation of Mo into the materials significantly improved the conversion rates, reaching 84.9% for CMo3 and 92.1% for CSn3Mo3 after 6 h. This suggests that the nature of the acidic sites present in the materials played a more critical role in the reaction efficiency than their textural properties. For systems modified with Mo, the reaction produced not only 5-HMF but also intermediates of the retro-aldol pathway (lactic acid (AL), pyruvaldehyde (PYR), and glyceraldehyde (GAA), highlighting the importance of Lewis acid sites in the formation of these species. Additionally, organic acids, such as levulinic acid (LEV) and formic acid (AF), were also detected. In the reuse tests, the observed loss of catalytic activity was attributed to a reduction in the number of Brønsted acid sites and the formation of humins during fructose conversion.

## Graphical abstract

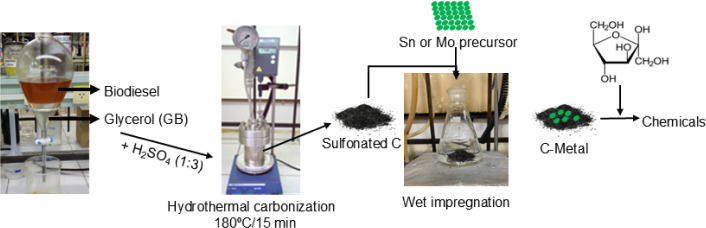

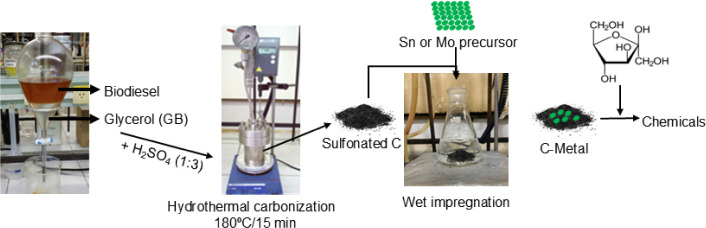

## 1 Introduction

In 2015, when the Sustainable Development Goals (SDGs) were established by the UN, 193 countries adopted the 2030 Agenda for development and sustainability.^1,2^ Many of the actions aimed at achieving the SDGs concern issues involving energy and renewable products. Brazil has enormous potential in generating renewable energy from biomass, and programs such as the production of bioethanol derived from sugarcane and biodiesel obtained from vegetable or animal oils and fats are consolidated examples that are part of the Brazilian and global energy matrix.^3,4^

Fructose is currently one of the main inputs for large-scale chemical synthesis,^5^ with emphasis on the production of 5-hydroxymethylfurfural (5-HMF) and other furanic compounds,^6,7^ such as levulinic acid (LEV) and formic acid (AF),^8,9^ in addition to 2-hydroxypropanoic acid, known as lactic acid (AL).^10–12^

In this context, heterogeneous catalysis has become essential for the processing of lignocellulosic materials and derivatives because of the possibility of obtaining dual systems containing sites of modulable nature and strength, which are associated with offering robustness and potential for recovery and reuse.^13–15^ In recent years, carbon-based compounds have been considered promising materials for the catalysis of biomass conversion because of their availability and low cost and because they can be obtained from residual biomass, which contributes to reducing the carbon footprint.^15,16^

Therefore, the conversion of several substrates, such as glucose, sucrose, maltose, lactose, mannitol, sorbitol, starch, and cellulose, in different solvents, such as water, methyl isobutyl ketone (MIBK), dimethylacetamide (DMA), γ-valerolactone (GVL), toluene, DMF, *n*-butyl alcohol, ethylene glycol, tetrahydrofuran (THF), sulfolane, 1,4-dioxane, ethanol, nitrobenzene, acetonitrile (ACT), and biphasic mixtures (water/DMSO and water/GVL), as well as saline solutions (DMSO/Na_2_SO_4_ and C_8_H_7_SO_3_Na), has been evaluated.^17–25^ However, the conversion of FRU in DMSO proved to be the most favorable condition described, and Table 1 illustrates a series of studies involving the conversion of fructose using various carbon-based solid catalysts, which employed mainly DMSO and water, the latter of which is used in the present study.^17,20,21,25^

**Table 1:** Examples of Studies Involving the Conversion of Fructose Using Various Carbon-Based Solid Catalysts in DMSO and Water^a^ ^b^ ^c^ ^d^

Catalytic system	Solvent	Catalyst amount (%)	Temp (°C)	Time (min)	Fructose conversion (%)	Yield of HMF (%)	Ref.
Activated carbon (AC) powder DARCO (Sigma-Aldrich) functionalized with p-toluene sulfonic acid (PTSA) (AC–PTSA)	DMSO^b^	11	120	120	95	92	^18^
Carbon sphere sulfonated (CS) generated from glucose	DMSO	20	160	150	98	90	^19^
Magnetic lignin residue-derived amorphous carbon sulfated (MLC–SO_3_H)	DMSO	50	130	40	100	81	^20^
Tobacco stem-derived porous carbon sulfated (S–TsC)	GLV/H_2_O	50	130	30	100	93.7	^21^
Phosphorus-doped graphitic carbon nitride (P–UCN)	DMSO	50	130	180	97.7	91.7	^22^
Solid acid (carbon) functionalized with oxalic acid (CC–oxa)	DMSO^a^	3	130	5	84.2	79.9	^23^
Carbonaceous obtained from sugar cane bagasse (SB) loaded with molybdenum (MC)	DMSO	5	120	120	100	85	^31^
Carbon obtained by carbonization and *in situ* sulfonation obtained from glycerol (CG)	DMSO	10	100	180	69	53.2	^17^
Lignin-derived sulfonated carbon (LDSC)	DMSO	5	100	60	49.9	40.6	^32^
Sulfonated carbon obtained from Eucalyptus Kraft Lignin (EKLSC)	DMSO^a^	15	120	0.167	93.3	91.4	^33^
Sulfonated carbon obtained from lignin (LCC)	DMSO/[BMIM][Cl]^a,c^	100	110	10	98	84	^24^
Phosphorylated mesoporous carbon (PMCs)	H_2_O^d^	50	120	960	78	53	^25^

a Microwave-assisted.

b Microwave prehomogenization.

c Butyl-3-methylimidazolium chloride (ionic liquid).

d Pressurized at 300 kPa of N_2_.

Charcoal and semicarbonized materials (biochar and hydrochar) have a high content of oxygenated groups, which facilitates the insertion of active groups on their surface, producing materials with distinct properties.^26,27^ Among the different types of materials, including amorphous sulfonic or sulfonated carbocatalysts (CSs), materials that have SO_3_H sites are low-cost protonic solid acid materials that are acidic and often comparable to concentrated H_2_SO_4_.^28^

Currently, several types of biochar/metal-based solid acid catalysts are being produced for the degradation of cellulosic materials.^29^ Rusanen et al. produced active carbons from birch sawdust residues that were functionalized with sulfonic groups and zinc species in different proportions. These materials were used in the conversion of glucose into 5-HMF in a two-phase system (water/THF) at 160 °C. The best results were obtained with the combination of sulfonated sites and Zn, with yields of 51% and a selectivity of 78% for 5-HMF.^30^

Despite the availability of research reports on metal-modified biochars, which are applied mainly as carbon monoxide adsorbents, effluents, supercapacitors, catalysts, and catalytic supports, there is great potential for innovation and the use of biochar produced from waste material for biomass conversion.^34–39^

From this perspective, this research aims to modify sulfonated carbon-based materials derived from residual glycerol from biodiesel with metallic species (Sn and Mo), characterize these materials, and assess their performance as heterogeneous catalysts in the conversion of fructose into industrially valuable products using water or DMSO as solvents. The proposed modification is expected to enhance the catalytic efficiency of these materials, making them more effective for fructose conversion into high-value industrial products.

## 2 Results and Discussion

Materials based on sulfonated C (C) modified with Sn or Mo, namely, CSn*x* (*x* = 3, 6, 9, and 12 wt % Sn), CMo3, and CSn3Mo3 (where Mo3 corresponds to the material with 3 wt % Mo), were synthesized, and their textural and physicochemical properties were characterized. Afterward, these materials were investigated as catalysts for the conversion of FRU in aqueous or organic media (DMSO), and both the catalytic efficiency and the yield of products formed were reported.

### 2.1 Characterization of C, CSn*x*, CMo3, and CSn3Mo3

To determine the chemical composition, CHN elemental analysis and inductively coupled plasma spectroscopy (ICP-OES) were carried out on the samples (Table 2). The results strongly indicate the successful modification of the biochar, as the presence of Sn and Mo was detected in the modified samples. The presence of S can be explained by the C synthesis method, which uses crude glycerol (a biodiesel byproduct) and H_2_SO_4_, resulting in the presence of sulfonated groups in the material.^28,40^ For the CSnx materials, the detected Sn content indicates a tendency to increase as the amount of precursor used in the synthesis increases (Table 2). For the CSn3Mo3-modified material, a higher Sn content was detected than Mo content (8.73% and 4.92% for Sn and Mo, respectively), despite the use of equivalent amounts of both metals during synthesis. This suggests stronger C–Sn interactions than C–Mo interactions, and similar trends have been observed in other studies where biochar was modified with metal species, indicating the establishment of stronger bonds between the carbon and Sn.^41^

**Table 2:** Chemical Composition and Textural Properties of C, CSnx, CMo3, and CSn3Mo3^a^ ^b^ ^c^ ^d^ ^e^ ^f^ ^g^

	C	CSn3	CSn6	CSn9	CSn12	CMo3	CSn3Mo3
C (%)^a^	63.45	67.72	66.91	61.35	64.73	69.32	53.52
H (%)^a^	4.95	2.57	4.04	3.26	1.91	3.66	1.56
N (%)^a^	0.09	0.16		0.19		0.57	nd
S (%)^b^	0.91	0.42	0.39	0.43	0.42	0.35	0.35
Sn (%)^b^	nd	1.13	1.72	2.11	1.97	nd	8.73
Mo (%)^b^	nd	nd	nd	nd	nd	0.98	4.92
*S*_BET_^c^ (m^2^ g^–1^)	46.8	29.3	9.1	29.2	26.6	67.7	43.8
*Vp*^d^ (cm^3^ g^–1^)	0.0467	0.0296	0.0167	0.0333	0.0259	0.0592	0.0368
*Dp*_BJH_^e^ (Å)	39	39	40	44	39	34	33

a Determined by CHN.

b Determined by ICP-OES.

c *S*_BET_, BET specific surface area.

d *Vp*, pore volume.

e *Dp*_BJH_, pore diameter.

f The values of *S*_BET_, *Vp*, and *Dp*_BJH_ were calculated by the BET and BJH equations.

g nd = not detected.

Still, it is important to mention that, according to the literature, biochars typically possess a high surface area and a negative surface charge, which makes them excellent sorbents for metal species, such as oxides. This high sorption capacity arises from specific adsorption on oxygenated functional groups (such as carboxyl and hydroxyl groups), electrostatic attraction to aromatic structures, and the precipitation of metal oxides on the mineral ash components of the biochar.^42^

The FTIR spectra (Figure 1) displayed bands between 1030 and 1175 cm^–1^, corresponding to the presence of sulfonic groups (SO_3_ stretching modes and symmetric stretching of O=S=O groups, respectively).^43^ In the C material, these absorptions appear more intense compared to the modified materials, suggesting a loss of sulfonic groups due to the impregnation process. The same behavior was observed at 1701 cm^–1^, corresponding to −C=O and −COOH groups, since the corresponding absorption band shows a slight decrease in intensity in the modified materials, indicating the degradation of some oxygenated groups during the modification stages. Additionally, the detection of a band at 1587 cm^–1^ indicates the presence of C=C bond absorption.^43^

**Figure 1:**
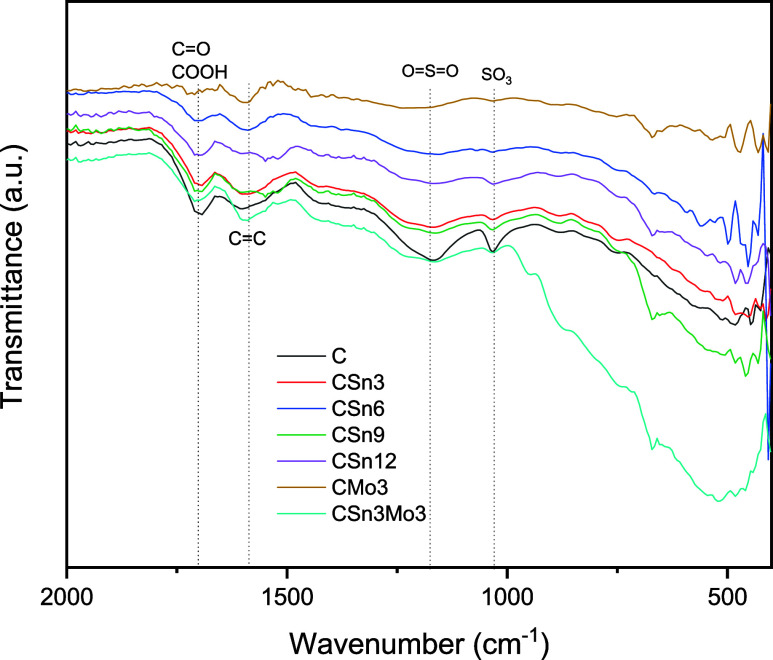
FTIR spectra of C, CSn*x*, CMo3, and CSn3Mo3.

In Figure 2A,B, the thermogravimetric profiles of the catalysts are shown, and the mass loss up to 100 °C for the C material (∼12%) and CSnx (∼8.4 to 9.7%) can be attributed to dehydration and the formation of volatile gases. Compared with the C material, the CSnx, CMo3, and CSn3Mo3 materials exhibited greater thermal stability in the range of 100–350 °C. For carbon-based materials, the mass loss in the 200–350 °C range is attributed to the degradation of carboxylic, sulfonic, and other oxygenated groups present.^44,45^ Notably, the mass loss within the degradation range of oxygenated or sulfonated groups is more pronounced for the metal-free material (6.3% mass loss) than for the other functionalized materials (CSn3 = 1.9%, CSn6 = 2.1%, CSn9 = 1.6%, CSn12 = 2.7%, CMo3 = 1.6%, and CSn3Mo3 = 2.0%). The TG/dTG results provide evidence of changes in the material composition after impregnation with Sn and/or Mo. Furthermore, the mass loss at temperatures above 400 °C is attributed to the graphitization processes of the polymeric structure of the C material (C = 22.0%, CSn3 = 21.2%, CSn6 = 20.2%, CSn9 = 20.0%, CSn12 = 20.5%, CMo3 = 23.0%, and CSn3Mo3 = 17.9%).^44,45^

**Figure 2:**
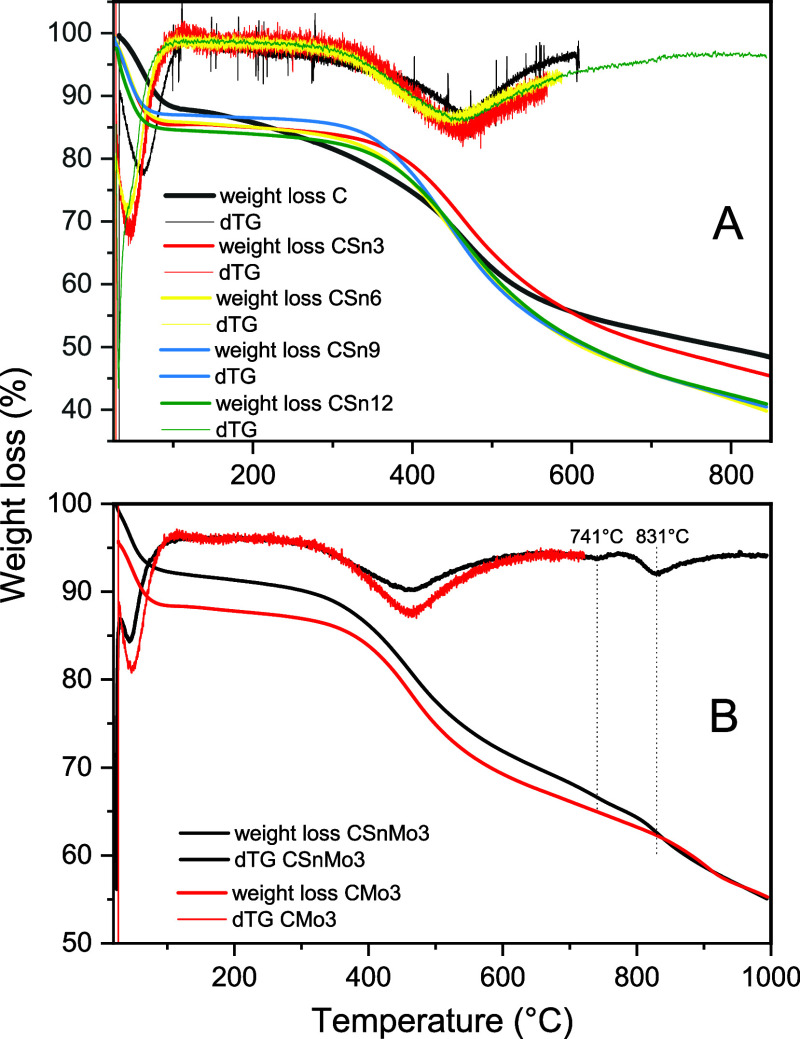
Thermal profiles (TG/dTG) of C and CSn*x* (A) and of CMo3 and CSn3Mo3 (B).

In Figure 2B, we observe a thermal event only in the materials modified with Mo. According to the dTG values obtained for CSn3Mo3 and CMo3, phenomena were detected at 831 and 850 °C, respectively. Typically, mass losses in this temperature range are attributed to the decomposition of molybdates and polymolybdates, leading to the formation of molybdenum oxide (MoO_3_).^46^ The fact that these losses occur at a higher temperature for the CMo3 material suggests stronger C–Mo interactions in this case.

The XRD patterns obtained for C (Figure 3) present characteristics of an amorphous material, according to the broadened reflection peak close to 2θ = 25°, which is characteristic of noncrystalline structures.^40^ For CSnx materials (Figure 3B–E), with increasing Sn content, a gradual increase in signal intensity is observed, and this phenomenon becomes more pronounced for CSn12 (Figure 3). In this context, peaks at 2θ = 26°, 33°, and 51° (corresponding to the (110), (101), and (211) planes, respectively) can be indexed, matching the three highest-intensity signals from the JCPDS No. 41-1445 crystallographic card, which corresponds to a tetragonal SnO_2_-type material with the *P*42/*mnm* space group. Furthermore, the broad signal between 2θ = 61° and 64° can be assigned to the (310), (211), (112), and (301) planes, respectively.^47–50^

**Figure 3:**
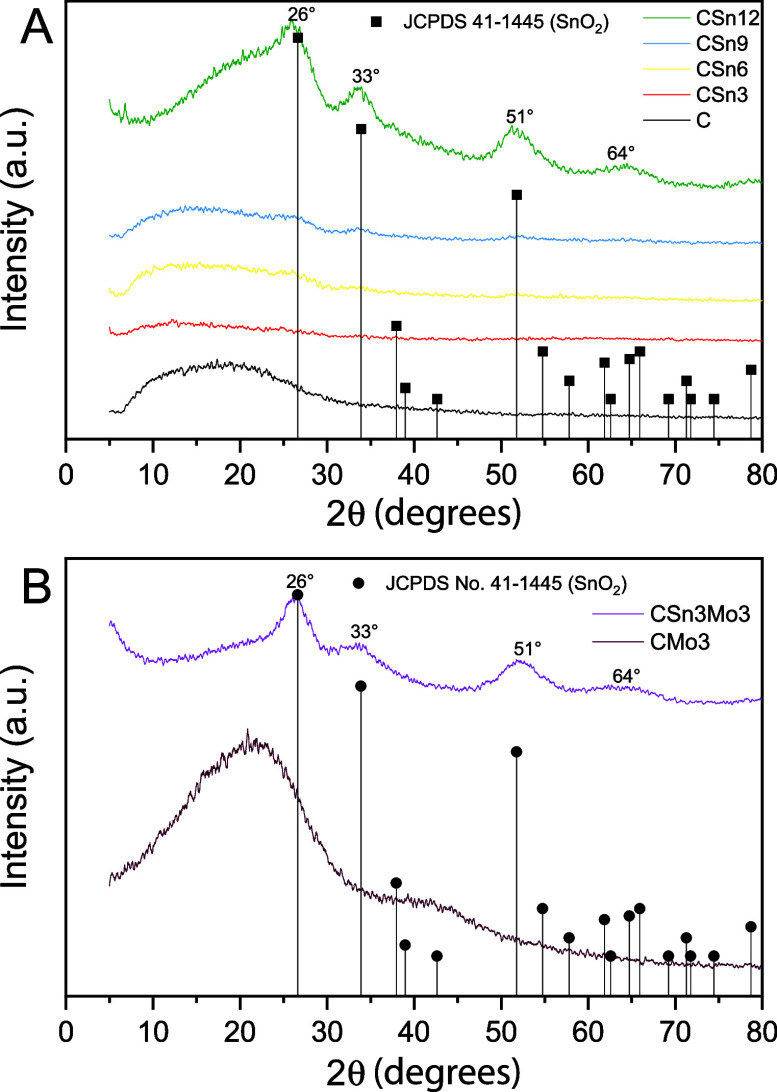
XRD patterns for C and CSn*x* (A); CMo3 and CSn3Mo3 (B).

For the CMo3 material, a broadened signal is present at 2θ = 45° in addition to the C signal at 2θ = 26°, which may indicate the presence of Mo species (Figure 3A). For the CSn3Mo3 material (Figure 3B), peaks such as those obtained with CSn12 (Figure 3A) were observed but were more intense, which suggests the formation of a more crystalline material, even though a 4-fold lower concentration of Sn was used than in the synthesis of CSn12. Thus, a synergistic effect may occur due to the presence of Sn and Mo metals, which causes an increase in the number of crystalline phases.^47,48,50^ Previous studies evaluated a series of SnO_2_-based catalysts modified with different concentrations of MoO_3_ without the use of biochar, and from the data obtained via UV–vis diffuse reflectance spectroscopy (DRS), it was possible to observe shifts in absorption to wavelengths close to the red spectrum (400 nm), mainly when the MoO_3_ content was increased. This behavior suggests the appearance of additional electronic levels with reduced bandgap energies.^50^ This phenomenon can explain the signal intensity and the possible Sn–Mo interaction effect in the biochar structure, which is supported by the decomposition of molybdate species observed in the TG–dTG characterizations (Figure 3B), mainly for the bimetallic catalyst.

Figure 4 shows an open hysteresis loop for all the isotherms, which can be attributed to several factors, such as material characteristics, the raw material source, or the synthesis method.^41^ This behavior corresponds to materials with a disorganized topology, complex and irregular structures, and noninterconnected pores, leading to the emergence of open micropores and other pores that are difficult to access. As a result, desorption from open pores occurs first, whereas the more inaccessible pores remain filled with adsorbate, even at low pressures, leading to an open hysteresis loop.^40,51^

**Figure 4:**
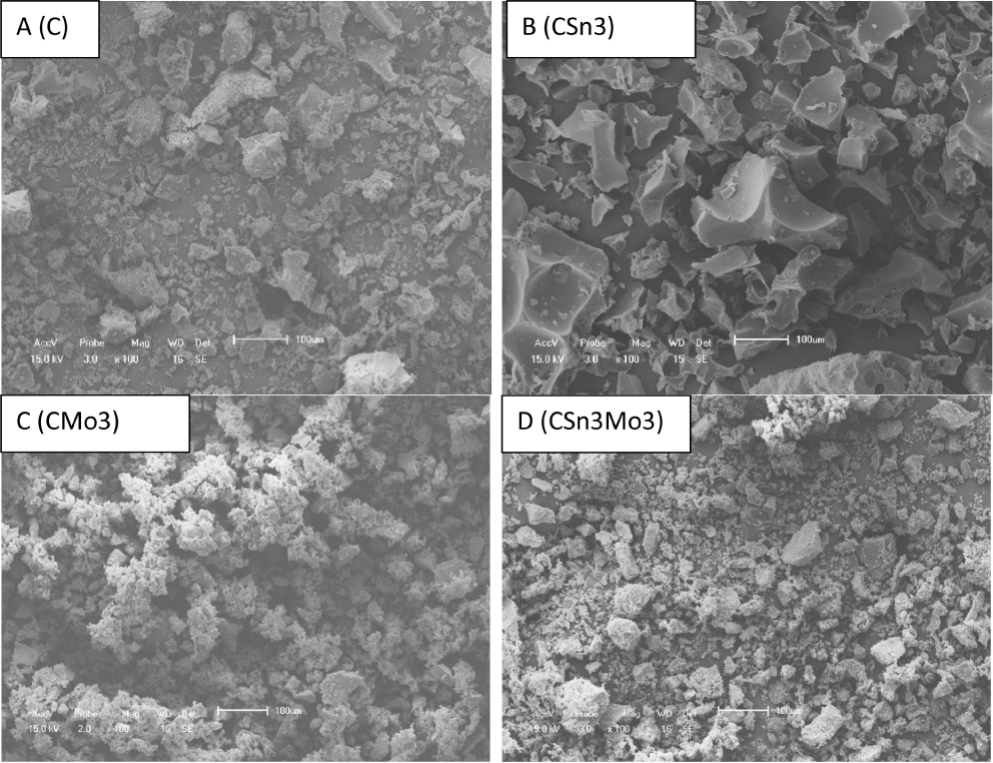
SEM images for C (A), CSn3 (B), CMo3 (C), and CSn3Mo3 (D).

Table 2 and Figure S1A–D present the specific surface areas, pore size distributions, and average pore sizes. The surface area of C was 46.8 m^2^ g^–1^, which is considered low for synthesized carbons.^52^ The use of raw biomass, such as the C obtained from crude glycerol from biodiesel, generally leads to the formation of materials with smaller surface areas due to pore blockage by coke formation, noncarbonized materials, and semidecomposed polymers.^41^ Another factor contributing to the lower surface area of C, compared with other carbonaceous species, could be the low carbonization temperature (180 °C in this study) and the fact that the material did not undergo activation, which is conventionally employed to produce activated carbons.^17^ Furthermore, the average pore size indicates the presence of mesopores, and it was similar for all of the materials. However, the pore diameter with the largest volume (∼39 Å) is close to the micropore range.^53^ The addition of 3, 9, and 12% Sn caused a reduction in surface area (29.3, 29.2, and 26.6 m^2^ g^–1^, respectively), a trend that can be explained by the impregnation of metal species in the C pore matrix.^54^ This behavior also contributes to the lower pore volume observed for the modified materials (Table 2).^55,56^

However, the material modified with Mo had a greater surface area than both the C- and Sn-modified materials, with areas of 68 and 44 m^2^ g^–1^ for CMo3 and CSn3Mo3, respectively. This increase in surface area is accompanied by an increase in pore volume, suggesting the formation of a greater pore network in the material. This could be due to the formation of complexes between metal species and oxygenated, sulfonated, or nondegraded polymeric functional groups during synthesis, which, after their removal, contribute to an increase in surface area.^41^

SEM (field-emission scanning electron microscopy) analysis (Figure 4A–D) with EDX (energy-dispersive X-ray spectroscopy) elemental mapping (Figure S2) was performed to investigate the surface morphology and composition of the materials. SEM images revealed a random morphology of carbon (C), exhibiting particles of different sizes, indicating a lack of specific patterns or structure in their surface appearance. In the case of CSn3, the image suggests that the addition of tin led to the formation of larger irregular particles compared to those observed for carbon. For CMo3, smaller and aggregated irregular particles are formed. The addition of Sn and Mo (CSn3Mo3) suggests the formation of particles with an appearance intermediate to those observed for CSn3 and CMo3. For the latter, SEM-EDX analysis shows a uniform coating of Mo or Sn, indicating that the metal species are uniformly distributed on the carbon surface.

### 2.2 Catalytic Essays in Water and DMSO

To evaluate the influence of the presence and amount of Sn, C, CSn3, CSn6, CSn9, and CSn12 (1.5 × 10^–3^ g) were tested for fructose conversion at 150 °C, employing water as the solvent, for 6 h (Figure 5).

**Figure 5:**
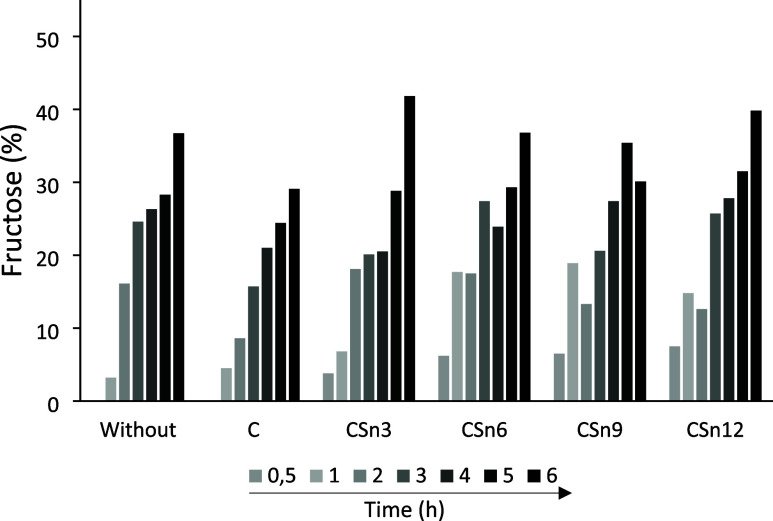
Fructose conversion (%) at 150 °C until 6 h, aqueous medium (solution of fructose = 0.044 mol L^–1^), without catalyst and employing 1.5 × 10^–3^ g of C, CSn3, CSn6, CSn9, and CSn12.

The use of the materials investigated did not lead to significant conversions, as it is possible to verify that even in the absence of a catalyst, a fructose conversion rate of 36.7% is observed under these conditions. The modification of C with different Sn contents did not have a major effect on the conversion rate. However, the CSn3 system can be highlighted, which, despite having the lowest Sn content compared with the other systems, leads to a slightly better result, with a conversion rate of 41.8% at 6 h. This behavior suggests that the maintenance of many Brønsted acid sites preexisting in C, as indicated by the FTIR results when lower levels of Sn are used, plays a fundamental role in the conversion of fructose.^57^

However, comparing the selectivity (%) of the different soluble products formed in the presence of C and CSn3 and in the absence of a catalyst (Figure 6) revealed that, in the absence of a catalyst or when C was used, selectivity to HMF was observed, which was more expressive when C was used, and this behavior can be related to the presence of Brønsted sites (sulfonic and oxygenated groups) in these materials.

**Figure 6:**
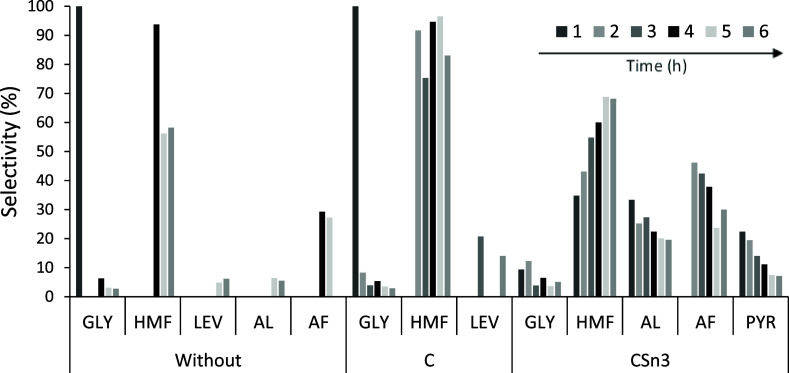
Amount (%) of soluble products formed at 150 °C until 6 h, aqueous medium (solution of fructose = 0.044 mol L^–1^), without catalyst and employing 1.5 × 10^–3^ g of C and CSn3.

However, in the presence of CSn3, in addition to the formation of 5-HMF, a significant amount of AL or intermediates of the retro-aldol pathway is observed (mainly pyruvaldehyde (PYR)), which can be explained by the presence of Lewis acid sites formed due to the presence of Sn. This result is very interesting because even if the presence of Sn does not lead to greater conversions, the modulation of the type of acidic site plays a fundamental role in the fructose conversion pathways for the formation of different products.^46^ Importantly, the nature and number of Lewis and Brønsted acid sites present in the catalyst and the synergism resulting from their combination are responsible for promising results in terms of converting carbohydrates into molecules of interest.^58–60^

5-HMF is formed by fructose dehydration with a consecutive loss of three water molecules. Nevertheless, owing to its high instability in aqueous media, organic acids, as well as humins, are formed, and this decomposition is favored in the presence of catalytic acidic sites,^46,61–65^ which also justifies the detection of AF via CSn3.

In this scenario, to modulate the acidic characteristics of the system, the CSn3 material was modified with Mo, since the formation of a bimetallic catalyst from these metals can have outstanding results in fructose conversion reactions due to the synergy between the weak Lewis acidity of Sn and the moderate to strong Lewis and Brønsted acidity of Mo.^46,49^ Figure 7 shows the conversion results for the C, CSn3, CMo3, and CSn3Mo3 systems.

**Figure 7:**
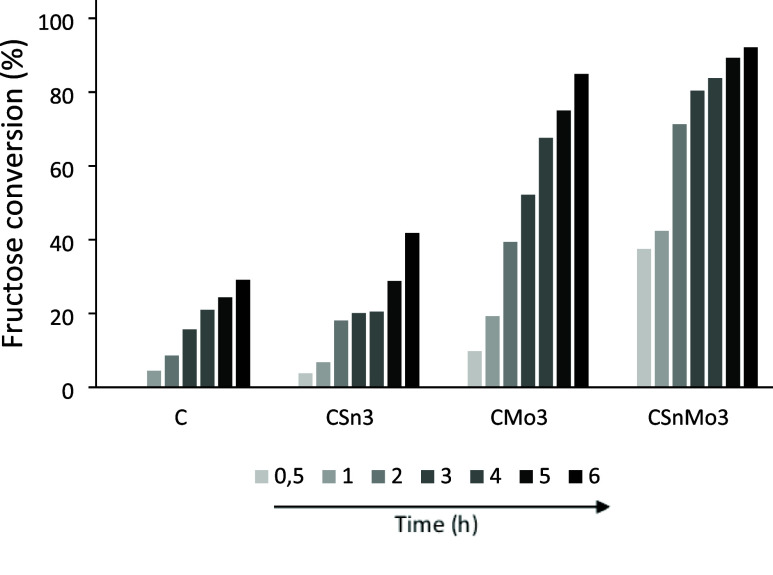
Fructose conversion (%) at 150 °C until 6 h, aqueous medium (solution of fructose = 0.044 mol L^–1^), employing 1.5 × 10^–3^ g of C, CSn3, CMo3, and CSn3Mo3.

The results clearly indicate that the presence of Mo in the materials led to higher conversion rates, reaching 84.9% for CMo3 and 92.1% for CSn3Mo3 after 6 h. These values cannot be directly attributed to the surface areas of these materials, which were 46.8, 29.3, 67.7, and 43.8 m^2^ g^–1^ for C, CSn3, CMo3, and CSn3Mo3, respectively, suggesting that the nature of the acidic sites present in the materials had a greater influence than their textural properties.

In terms of selectivity (Figure 8), the systems modified with Mo, in addition to being more active, also led to greater selectivity toward AL and other intermediates of the retro-aldol pathway (PYR and glyceraldehyde (GAA)), further confirming the importance of Lewis acidic sites for the formation of these species. Additionally, the presence of organic acids (LEV and AF), which are formed by the rehydration reaction of the 5-HMF produced in the presence of acidic catalysts, was observed.^3,66^

**Figure 8:**
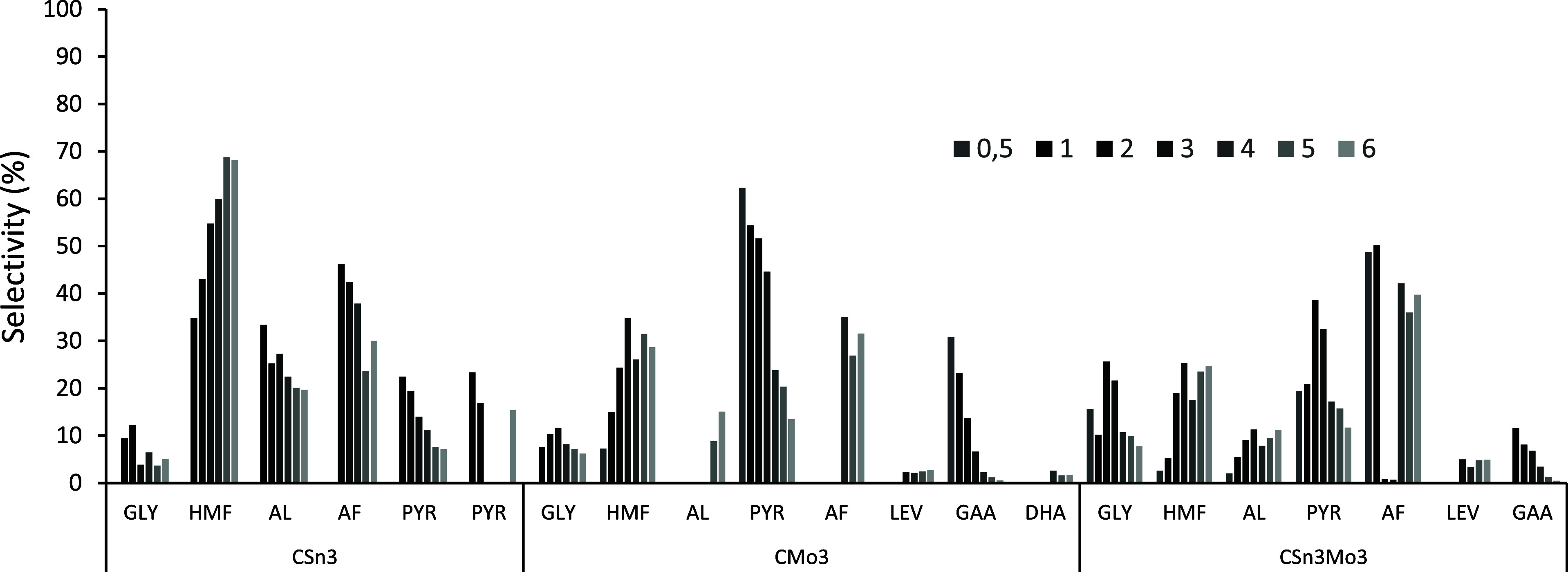
Amount (%) of soluble products formed at 150 °C until 6 h, aqueous medium (solution of fructose = 0.044 mol L^–1^), employing 1.5 × 10^–3^ g of CSn3, CMo3, and CSn3Mo3.

To evaluate this type of system further, C, CSn3, CMo3, and CSn3Mo3 catalysts were investigated in the presence of DMSO as the solvent at 120 °C (Figure 9). This temperature was chosen because of the greater activity of the systems in this reaction medium since at 150 °C (the temperature used in the studies in water), the total conversion of fructose was already observed early in the reaction. Reports indicate that DMSO acts by itself as a catalyst for this reaction^18^ and can also stabilize the 5-HMF produced, preventing its rehydration and the formation of AL and AF.^67–69^

**Figure 9:**
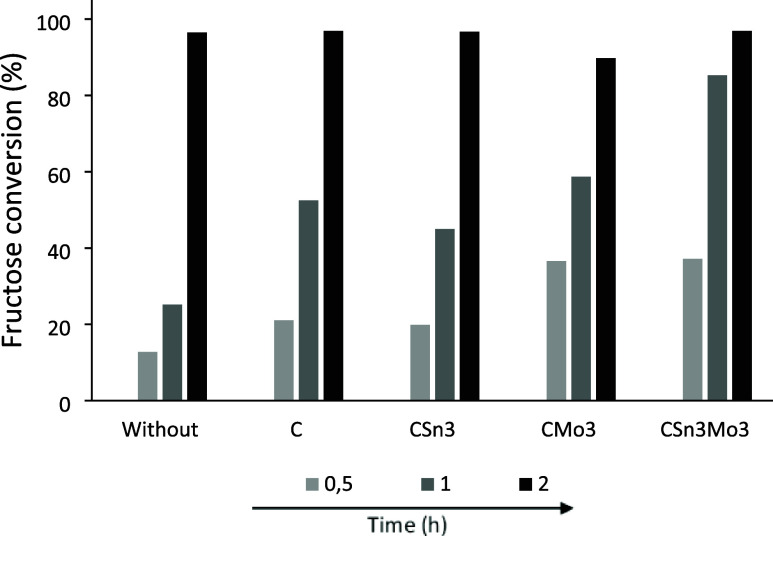
Fructose conversion (%) at 120 °C until 2 h, DMSO (solution of fructose = 0.044 mol L^–1^), without catalyst and employing 1.5 × 10^–3^ g of C, CSn3, CMo3, and CSn3Mo3.

First, within 2 h of reaction, the conversion is practically complete for the investigated systems. However, it is possible to compare the systems for up to 1 h, and as already observed in aqueous media, the high points are the systems containing Mo, which presented fructose conversion values of 58.7 and 85.3% for CMo3 and CSn3Mo3, respectively, at 1 h. C, which contains Brønsted acid sites, led to a 52.5% conversion.

The most prominent difference when DMSO is used as a solvent is the high selectivity of the systems for 5-HMF (between ∼80 and 93%), as shown in Figure 10, which probably results from the synergism between the action of DMSO (Brønsted acid) and the other Brønsted and Lewis acid sites present in the materials and from its ability to stabilize the 5-HMF formed, as previously described.^18–69^ The detection of LEV can be justified by the rehydration of 5-HMF, and the presence of GAA, a product of the retro-aldol pathway, occurs primarily because of the presence of Lewis acid sites on the materials; however, these formations occur to a small extent in the organic medium used.

**Figure 10:**
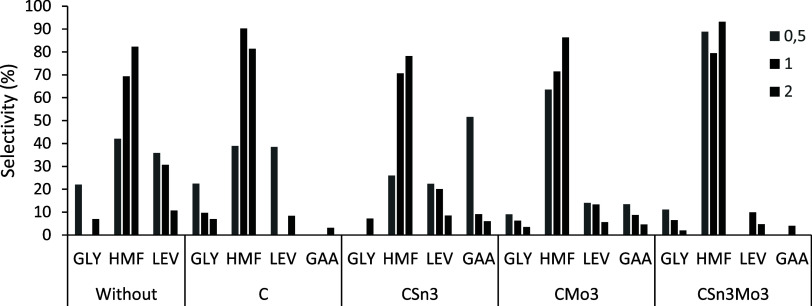
Selectivity (%) of soluble products formed at 120 °C until 2 h, DMSO (solution of fructose = 0.044 mol L^–1^), without catalyst and employing 1.5 × 10^–3^ g of C, CSn3, CMo3, and CSn3Mo3.

### 2.3 Reuse of the Catalyst CSn3Mo3

The catalyst recovery and reuse tests, employing CSn3Mo3 (3 and 4 cycles in water or DMSO, respectively), at 150 and 120 °C, respectively, using 1.5 × 10^–3^ g of catalyst in 1 h, were investigated. The results point out a significant decline in catalytic activity in both reaction media (Figure 11), even with the catalyst washing and recalcination (300 °C) procedure between the cycles adopted in this work.

**Figure 11:**
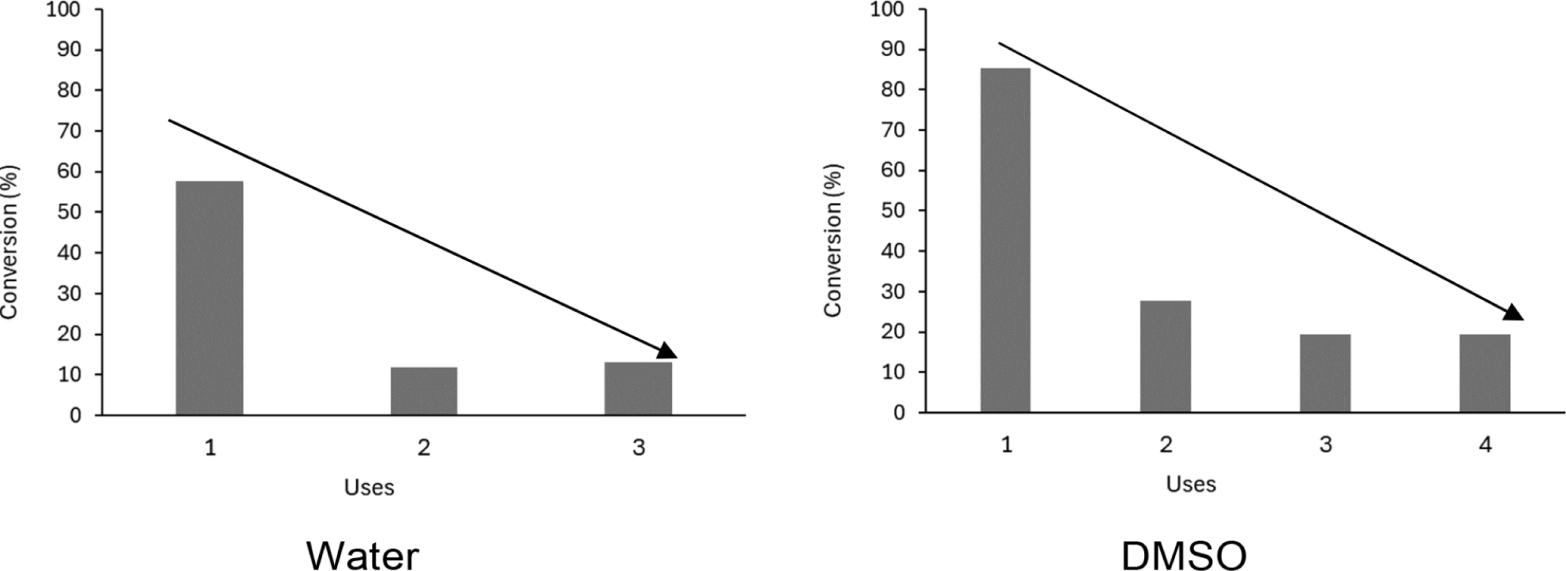
Reuse tests in fructose conversion (%), in water (solution of fructose = 0.044 mol L^–1^; 150 °C) and DMSO (solution of fructose = 0.044 mol L^–1^; 120 °C), employing 1.5 × 10^–3^ g of CSn3Mo3.

To obtain further clarification of the possible reasons that led to the loss of activity observed when CSn3Mo3 was used, this system and C were recovered after 6 h at 150 °C for water and 2 h at 120 °C for DMSO, maintaining the other reaction conditions that were already being used.

Initially, the chemical composition results obtained by CHN and ICP analyses (Table 3) indicate that in the case of CSn3Mo3 used in an aqueous medium there is a loss of S, which may be associated with the degradation or removal of sulfonic groups. This behavior is not observed in DMSO, possibly due to the presence of residual solvent, which may have increased this value. This trend is confirmed when analyzing the FTIR spectra, in which a decrease in the absorption bands related to these groups is observed for both C and CSn3Mo3 after reuse with water (Figure 12).

**Table 3:** Chemical Composition for CSn3Mo3 before and after Reuse in Water and DMSO

	CSn3Mo3 before use	CSn3Mo3 reuse H_2_O	CSn3Mo3 reuse DMSO
C (%)^a^	53.52	50.27	48.40
H (%)^a^	1.56	2.21	2.20
N (%)^a^	nd	0.31	0.32
S (%)^b^	0.35	0.25	0.56
Sn (%)^b^	8.73	9.09	9.11
Mo (%)^b^	4.92	4.88	6.01

**Figure 12:**
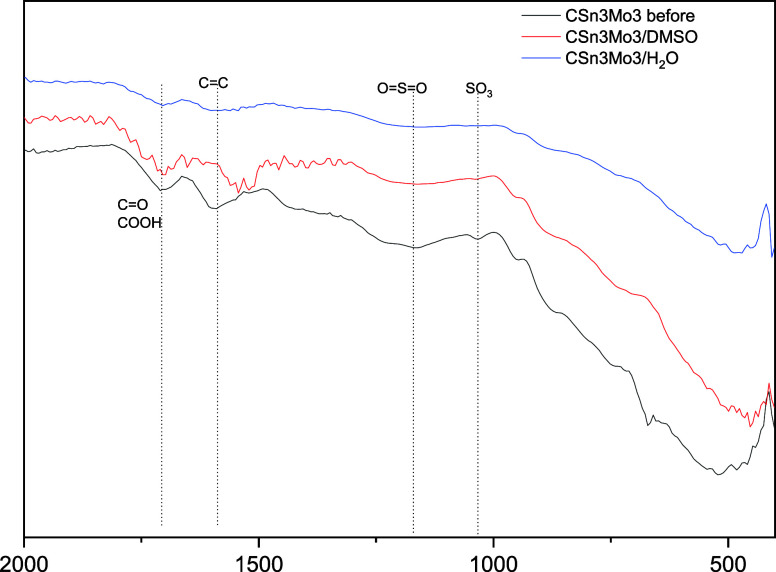
FTIR spectra of CSn3Mo3 before and after reuse.

Additionally, the XRD diffractograms for CSn3Mo3 (Figure S3) do not indicate significant structural modifications, suggesting that no significant losses of Sn or Mo species occurred, confirming what had already been detected by elemental analysis.

The set of results suggests that the loss of Brønsted acid sites contributed in part to the loss of activity but does not fully justify it, since the Sn and Mo species remained on the surface of the C. It is important to highlight that reports in the literature suggest that the loss of activity of this type of material may also be associated with the formation of humins during the conversion of fructose, followed by their entrapment due to the textural properties of the systems.^57^ In this sense, the visual aspect of some reactions carried out here (Figures S4 and S5) confirms the formation of insoluble humin-type materials and soluble oligomeric materials, which give color to the solutions. Probably, these materials were not completely eliminated using calcination at 300 °C, a process carried out between each cycle.

Additionally, the investigation of the thermal behavior of the CSn3Mo3 catalyst, before and after reuse, confirms an increase in mass loss for the catalyst after its use in the fructose conversion reaction in an aqueous medium (Figure S6). For example, at temperatures between 300 and 700 °C, the mass loss was 4.5% higher than that observed for the unused catalyst, constituting a strong indication that this phenomenon indicates the elimination of the humins formed.^70^

## 3 Conclusions

Sulfonated carbon-based materials modified with metallic species (Sn and Mo) proved to be efficient catalysts for fructose conversion in water or DMSO, as their use led to higher conversions compared to those observed without a catalyst or in the presence of C. The presence of Mo was crucial in enhancing conversion rates, with highlights for CMo3 (84.9%) and CSn3Mo3 (92.1%) after 6 h of reaction. These results indicate that the nature of the acidic sites plays a more significant role in the reaction efficiency than the textural properties of the materials. In addition to producing 5-HMF, the catalysts modulated with Mo facilitated the formation of intermediates of the retro-aldol pathway (AL, PYR, and GAA) and organic acids (LEV and AF), emphasizing the importance of Lewis acid sites in this process. However, the reduction in catalytic activity during reuse tests was attributed to a decrease in Brønsted acidic sites and the formation of humins. These findings underline the potential of these materials as catalysts while highlighting the need for strategies to enhance their stability and reusability.

## 4 Experimental Section

### 4.1 Materials

Fructose P.A. 99% Sigma-Aldrich (FRU), sulfuric acid conc. P.A. 99% Dinâmica H_2_SO_4_, tin tetrachloride pentahydrate 99% Sigma-Aldrich SnCl_4_·5H_2_O, ammonium heptamolybdate tetrahydrate ((NH_4_)_6_Mo_7_O_24_·4H_2_O) Sigma-Aldrich; DMSO (dimethyl sulfoxide) from Sigma-Aldrich was used as received.

### 4.2 Synthesis and Characterization of Catalysts

The carbon-based heterogeneous catalysts were obtained from glycerol-derived carbon through hydrothermal semicarbonization in a stainless-steel autoclave reactor at a temperature of 180 °C for 15 min, in the presence of H_2_SO_4_ (2%) as a polymerizing and sulfonating agent. Subsequently, the carbonized material (C) was washed with water/acetone and dried for 1 day at 60 °C.^52,56^ The Sn impregnation steps were performed via wet impregnation, using excess solutions containing 3%, 6%, 9%, and 12% (wt %) of metal, with SnCl_4_·5H_2_O as the precursor.^57^ The suspension of C and the precursor solution were slowly mixed and stirred for 24 h at room temperature. Then, the material was filtered (quantitative filters), washed with distilled water, and dried at 60 °C for 1 day. Finally, the collected material was subjected to calcination for 4 h in an oxygen-free atmosphere at a temperature of 300 °C with a heating ramp of 10 °C/min. The resulting materials were named CSnx (CSn3, CSn6, CSn9, and CSn12, according to the respective metal contents in the precursor solutions). Additionally, materials modified with Mo were produced using (NH_4_)_6_Mo_7_O_24_·4H_2_O as the precursor, as well as a mixture of (NH_4_)_6_Mo_7_O_24_·4H_2_O and SnCl_4_·5H_2_O for the production of the bimetallic material, all maintaining 3% metal contents in the precursor solutions. These were named CMo3 and CSn3Mo3, respectively.

The materials were characterized via thermogravimetric analysis (TG/dTG), energy-dispersive X-ray spectroscopy (EDX), Fourier transform infrared spectroscopy (FTIR), physisorption (BET), and X-ray diffraction (XRD), among others. Thermogravimetric data were obtained using TRIOS equipment from TA Instruments, with a heating range from room temperature up to 850 °C at a rate of 10 °C/min and nitrogen as a carrier gas at 50 mL/min. Nitrogen physisorption analyses were performed using a gas adsorption analyzer from Quantachrome, model NOVA 2200e, with nitrogen at ∼77 K (−196 °C) as the adsorbate. The pretreatment of the samples was carried out for 24 h at 100 °C under vacuum. The Brunauer–Emmett–Teller (BET) method was used to determine surface area, while the Barrett, Joyner, and Halenda (BJH) model was employed to determine pore size and distribution.^46^ The Sn, Mo, and S content was determined using a Spectro Arcos ICP-OES (inductively coupled plasma optical emission spectrometry) optical spectrometer (Kleve, Germany). The samples were digested with a 30% HNO_3_:HF:H_2_O_2_ mixture in a 1:2:0.5 ratio in closed bottles in a digester block for 1 h at 100 °C. Fourier transform infrared spectroscopy (FTIR) was performed by using a Nicolet 6700 FTIR spectrophotometer. Samples were dispersed in KBr for pellet formation or analyzed in ATR (attenuated total reflectance) mode. The scan parameters were in the spectral range of 400–4000 cm^–1^, measured in 64 scans in transmittance mode, with a resolution of 16 cm^–1^.^58^ X-ray diffraction (XRD) analyses were carried out using a Shimadzu XRD-6000 X-ray diffractometer. The experiments applied a 40 kV voltage with a current of 30 mA. Scans were conducted in 2θ intervals, ranging from 5° to 90°, with a step size of 0.02° and a speed of 2°/min. The powdered samples were subjected to a Cu Kα radiation source (1.5418 Å), using divergence and scattering slits of 1° and a receiving slit of 0.30 mm.^58^ Scanning electron microscopy (SEM) analyses were performed using a Shimadzu SSX-550 Superscan. Prior to imaging, the samples were coated with a thin layer of gold using a Sanyu Electron Quick Coater SC-701 sputter coater. The metallization process was carried out for 5 min with a current of 10 mA. Energy-dispersive X-ray spectroscopy (EDX) analyses were conducted at a magnification of 2000×.

### 4.3 Catalytic Tests

The reactions were carried out in 5 mL sealed vials with controlled temperature and stirring. FRU (0.016 g) was solubilized in 2 mL of deionized water or DMSO (0.044 mol L^–1^). Temperatures of 150 and 120 °C (for water and DMSO, respectively) were used, with a catalyst load of 1.5 × 10^–3^ g (9% relative to the mass of FRU) and reaction times ranging from 0.5 to 6 h. At the end, the catalyst was collected by centrifugation, and the samples were filtered (0.45 μm filters) for quantification. Finally, the reaction mixture was analyzed using High–Performance liquid chromatography (HPLC) for product identification.^29,49,59–62^

Fructose conversion was calculated according to eq 1, in which *C*(%) = fructose conversion; *C*_0_ = initial concentration of fructose (mol/L); and *C*_f_ = final concentration of fructose (mol/L).

1 $$C\left(\%\right)=\left(\frac{C_{0}-C_{f}}{C_{0}}\right)\times 100$$

The yield of each soluble product obtained and duly identified was calculated according to eq 2, in which *R*_*i*_ (%) = yield of product *i*; *C*_*i*_ = concentration obtained from product *i* (mol/L); *C*_0_ = initial fructose concentration.

2 $$R_{i}\left(\%\right)=\left(\frac{C_{i}}{C_{0}}\right)\times 100$$

The selectivity of each product was calculated according to eq 3, in which *S_i_* (%) = selectivity of product *i*; *C*_*i*_ = concentration of product *i*; *C*_*i*1_, *C*_*i*2_, *C*_*i*3_, *C*_*i*4_, and *C*_*i*5_ = concentrations of other products (mol/L).

$$S_{i}\left(\%\right)=\left(\frac{C_{i}}{C_{i1}+C_{i2}+C_{i3}+C_{i4}+C_{i5}}\right)$$ 3

## Supplementary Material



## Notes

SEM (field-emission scanning electron microscopy) analysis along with EDX (energy-dispersive X-ray spectroscopy) elemental mapping (Figure S1); textural properties (Figure S2); the XRD patterns of CSn3Mo3 after reuse (Figure S3); visual appearance of the samples during the reaction, both with and without catalyst, at 150 °C for periods ranging from 0.5 to 6 h (Figures S4 and S5); thermal profiles (TG/dTG) of CSn3Mo3 after reuse in water (Figure S6) (PDF)
