# Supplementary material for: Sn- and Mo-Modified Sulfonated Carbons: Properties and Evaluation as Catalysts for Fructose Conversion in Water and DMSO
Source: ACS Omega. 2025 May 29;10(22):22919–29. doi: 10.1021/acsomega.5c00450 (PMC12163813; doi:10.1021/acsomega.5c00450)
Supplement: Supplementary file 1 [file ao5c00450_si_001.pdf]

Sn- and Mo-modified sulfonated carbons: properties and evaluation as catalysts for fructose conversion in water and DMSO

Felyppe Markus Ribeiro Sobral Altino [1], Wander dos Santos Sá [1], Jailma Barros dos Santos [1], Wagner Alves Carvalho [2,], Simoni Margareti Plentz Meneghetti [1,*]*

Supplementary information

Figure S1 SEM (Field-Emission Scanning Electron Microscopy) analysis with EDX (Energy-Dispersive X-ray Spectroscopy) elemental mapping

Figure S2 N_2 adsorption–desorption isotherms for: C and CSnx (A), CMo3 and CSn3Mo3 (B), pore size distributions and average pore sizes for C and CSnx (C) and for CMo3 and CSn3Mo3 (D).

Figure S3 XRD patterns for CSn3Mo3 (before reaction and after reuse).

Figure S4 Visual aspects of the samples during the reaction, with and without the catalyst at 150 °C (0.5 to 6 h) in water.

Figure S5 Visual aspects of the samples during the reaction, using catalysts and without catalyst at 150 °C (0.5 to 6 h) in DMSO

Figure S6 Thermal profiles (TG/dTG) for CSn3Mo3 (before reaction and after reuse in water).

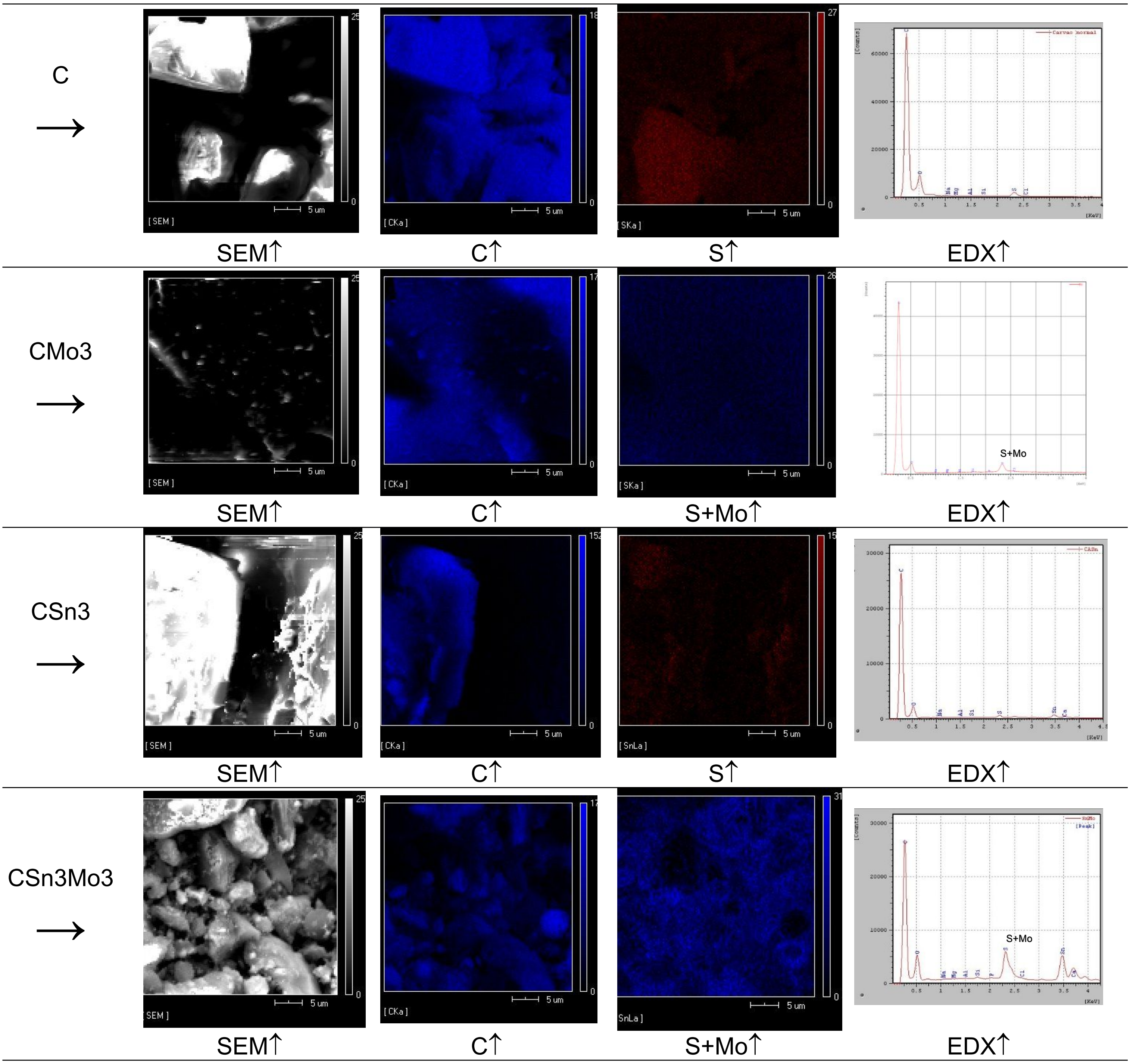

Figure S1 SEM (Field-Emission Scanning Electron Microscopy) analysis with EDX (Energy-Dispersive X-ray Spectroscopy) elemental mapping

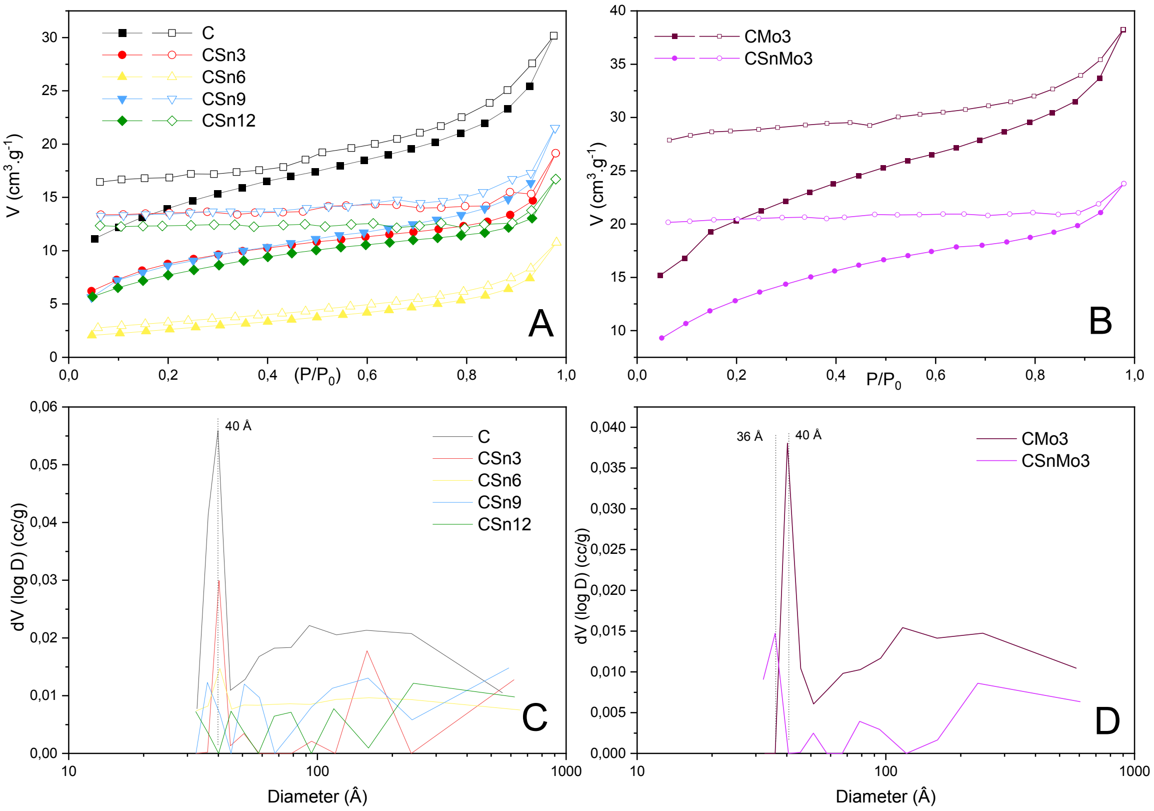

Figure S2 N_2 adsorption–desorption isotherms for: C and CSnx (A), CMo3 and CSn3Mo3 (B), pore size distributions and average pore sizes for C and CSnx (C) and for CMo3 and CSn3Mo3 (D).

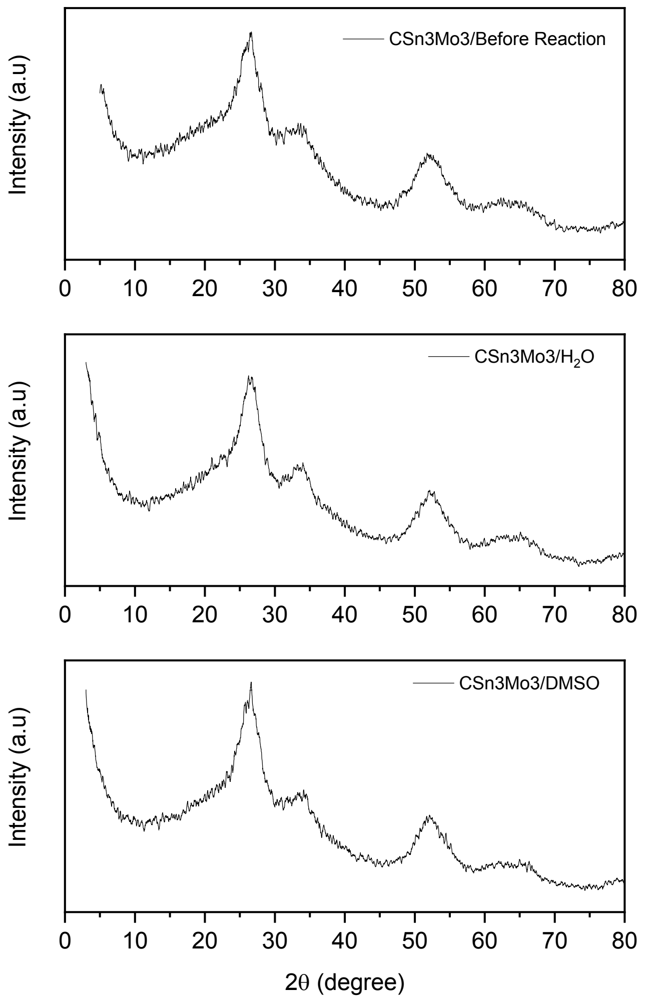

Figure S3 XRD patterns for CSn3Mo3 (before reaction and after reuse).

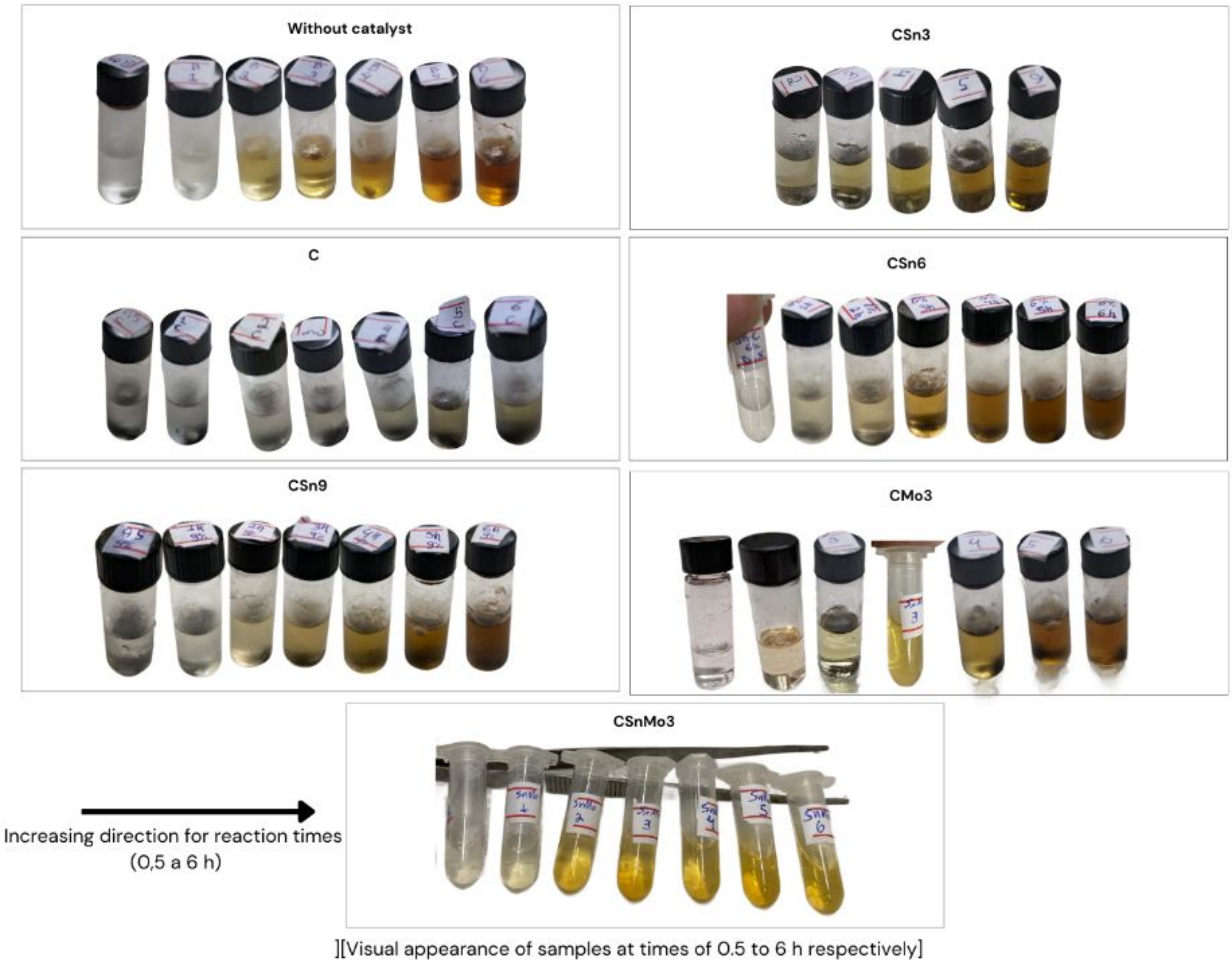

Figure S4 Visual aspects of the samples during the reaction, with and without the catalyst at 150 °C (0.5 to 6 h) in water.

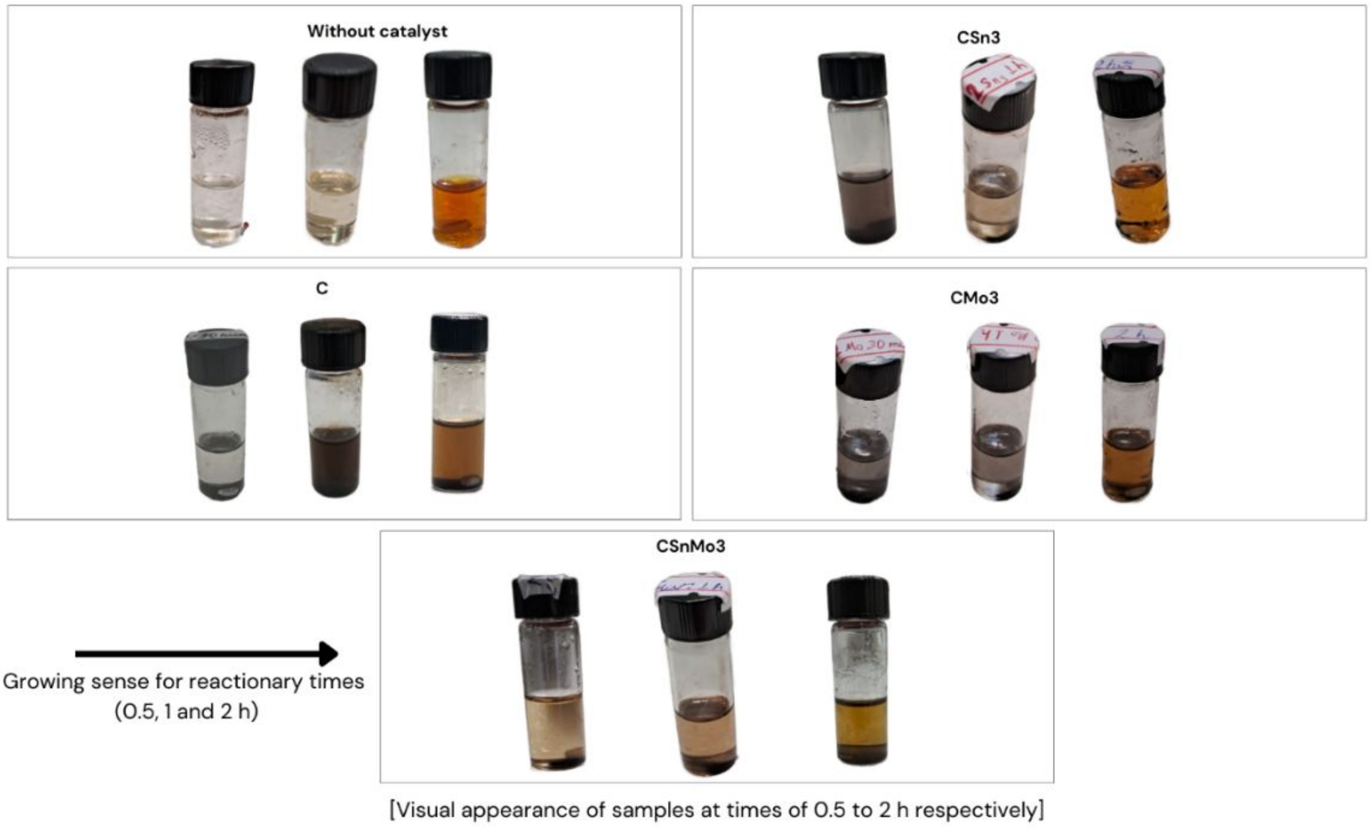

Figure S5 Visual aspects of the samples during the reaction, using catalysts and without catalyst at 150 °C (0.5 to 6 h) in DMSO

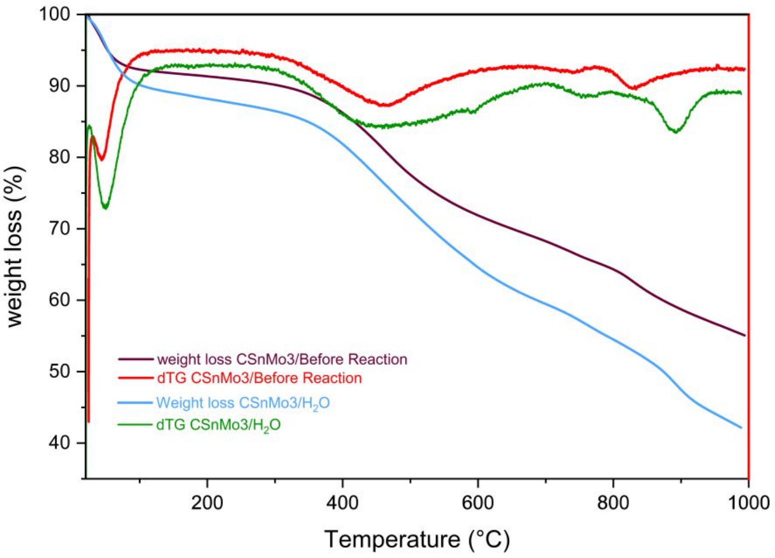

Figure S6 Thermal profiles (TG/dTG) for CSn3Mo3 (before reaction and after reuse in water).
